# Pathology and Morbid Anatomy

**Published:** 1901-09

**Authors:** 


### Books and Magazines.

---

Pathology and Morbid Anatomy. By T. Henry Green, M. D., F. R. C. P., Physician and Special Lecturer on Clinical Medicine at Charing Cross Hospital, etc. New (9th) American from ninth English edition. Revised and enlarged by H. Montague Murray, M. D., F. R. C. P., Lecturer on Pathology and Morbid Anatomy at Charing Cross Hospital; revised for America by Walton Martin, Ph. B., M. D., of the College of Physicians and Surgeons, New York City. Handsome octavo volume of 578 pages with four colored plates and 339 engravings. Cloth, $3.25 net. Lea Brothers & Co., Publishers, Philadelphia and New York.

Green's Pathology and Morbid Anatomy is a standard book the world over. It has now gone through nine editions American and English. This edition has been considerably enlarged and revised by H. Montague Murray, of London, lecturer on pathology and morbid anatomy at Charing Cross Hospital. The American revision is by Walton Martin, of the College of Physicians and Surgeons, New York City, who has supplied complete chapters on malaria and on the blood, and also a chapter on the preparation and staining of tissues for microscopic study. The style is strong and lucid, and the illustrations are modern and practical. It is the text-book in nearly all medical colleges. T. J. B.

